# Report on Ovaries Removed by Dr. T. G. Thomas

**Published:** 1874-06

**Authors:** 


*AMERICAN JOURNAL OF OBSTETRICS—May*, 1874:

Report of the Ovaries Removed by Dr. T. G. Thomas.—Dr. Noeggerath read a detailed account of the macro and microscopical examination made by him of the two ovaries removed by Dr. Thomas several months ago. The ovaries are of normal size, covered with numerous fine thread-like adhesions and filaments. Under the microscope both ovaries were found to be in a high degree of interstitial inflammation and fatty degeneration, the graafian follicles partly obliterated and partly filled with hemorrhagic clots. A number of bodies similar to, and probably pacinian corpuscles, with what appeared to be nervous filaments leading to them, were found in the ovaries, from the whole appearance of which it was evident that no treatment short of removal would have been of service.

In answer to a question from Dr. Jacobi, as to what he would infer from the pacinian corpuscles in the ovary, Dr. Noeggerath said that he did not draw any inference therefrom, except that there was fatty degeneration and the formation of new nerve-tissue. Pacinian corpuscles have been stated to have been found once in a hypertrophied cervix uteri, but there is no literature on the subject. He attributes the constant and agonizing pain experienced by the patient in the ovaries not to the fatty degeneration, but principally to the inflammation of the peritoneal envelop of the organ, and the consequent cicatrical contraction. This perimetritis may have been present since childhood, for he has seen a case in a child two and a half years of age.

Dr. Peaslee thought that, irrespective of the pacinian corpuscles, there was enough found in the ovaries to account for the distressing symptoms. As a rule, a woman who has had children will bear more pain in the ovaries, and think less of it than a virgin, in whom these cases of interstitial oophoritis are rarer than in married women.

Dr. Jacobi said that interstitial oophoritis is more common in women subject to frequent sexual excitement, such as street-walkers.

